# Meta-Analysis Identification of Highly Robust and Differential Immune-Metabolic Signatures of Systemic Host Response to Acute and Latent Tuberculosis in Children and Adults

**DOI:** 10.3389/fgene.2018.00457

**Published:** 2018-10-04

**Authors:** Saikou Y. Bah, Thorsten Forster, Paul Dickinson, Beate Kampmann, Peter Ghazal

**Affiliations:** ^1^Division of Pathway Medicine and Edinburgh Infectious Diseases, University of Edinburgh Medical School, Edinburgh, United Kingdom; ^2^West African Centre for Cellular Biology of Infectious Pathogens, University of Ghana, Accra, Ghana; ^3^Vaccines and Immunity Theme, Medical Research Council Unit The Gambia at the London School of Tropical Medicine and Hygiene, Banjul, Gambia; ^4^Centre of International Child Health, Department of Paediatrics, Imperial College London, London, United Kingdom; ^5^Systems Immunity Research Institute, School of Medicine Laboratory of Immunity and Metabolism, University of Cardiff, Wales, United Kingdom

**Keywords:** tuberculosis, meta-analysis, immunity, systemic responses, microarray, bioinformatics

## Abstract

**Background:** Whole blood expression profiling is a mainstay for delineating differential diagnostic signatures of infection yet is subject to high variability that reduces power and complicates clinical usefulness. To date, confirmatory high confidence expression profiling signatures for clinical use remain uncertain. Here we have sought to evaluate the reproducibility and confirmatory nature of differential expression signatures, comprising molecular and cellular pathways, across multiple international clinical observational studies investigating children and adult whole blood transcriptome responses to tuberculosis (TB).

**Methods and findings:** A systematic search and quality control assessment of gene expression repositories for human TB using whole blood resulted in 11 datasets with a total of 1073 patients from Africa, Europe, and South America. A non-parametric estimation of percentage of false prediction was used for meta-analysis of high confidence differential expression analysis. Deconvolution analysis was applied to infer changes in immune cell proportions and enrichment tests applied using pathway database resources. Meta-analysis identified high confidence differentially expressed genes, comprising 372 in adult active-TB versus latent-TB (LTBI), 332 in adult active-TB versus controls (CON), five in LTBI versus CON, and 415 in childhood active-TB versus LTBI. Notably, these confirmatory markers have low representation in published signatures for diagnosing TB. Pathway biology analysis of high confidence gene sets revealed dominant metabolic and innate-immune pathway signatures while suppressed signatures were enriched with adaptive signaling pathways and reduced proportions of T and B cells. Childhood TB showed uniquely strong inflammasome antagonist signature (*IL1RN* and *ILR2*), while adult TB patients exhibit a significant preponderance type I and type II IFN markers. Key limitations of the study include the paucity of data on potential confounders.

**Conclusion:** Meta-analysis identified high confidence confirmatory immune-metabolic and cellular expression signatures across studies regardless of the population resource setting, HIV status and circulating endemic pathogens. Notably, previously identified diagnostic signature markers for TB show limited concordance with the confirmatory meta-analysis. Overall, our results support the use of the confirmatory expression signatures for guiding optimized diagnostic, prognostic, and therapeutic monitoring modalities in TB.

## Introduction

Tuberculosis caused by members of the *Mycobacterium tuberculosis* complex is a leading cause of morbidity and mortality due to an infectious agent despite the availability of potent antimycobacterials (WHO, 2015). About a third of the world population is latently infected with *M. tuberculosis* with a life time risk of re-activation to cause active TB and transmission to other susceptible hosts. BCG, the only licensed TB vaccine has variable efficacy ranging from 0–80% with limited efficacy in preventing pulmonary TB in adults who are largely responsible for disease transmission (Colditz et al., 1994; Karonga Prevention Trial Group, 1996). The control of TB is further complicated due to the emergence of antimycobacterial resistance and co-infection with HIV, with TB as the leading cause of death in HIV co-infected patients (Bruchfeld et al., 2015). Furthermore, the host immune response to mycobacterial infection although thought to be primarily T cell mediated is very complex and remains incompletely understood (Cooper, 2009; Dwivedi et al., 2012) and there are currently no validated immune correlates of protection.

To develop the more efficacious vaccines identified as essential for global TB control, it is important to understand the host immune response to *M. tuberculosis.* A promising approach to interrogate the host response at a systemic level in order to identify correlates of protection is systemic analyses of host transcriptional responses, using methods such as microarrays and increasingly RNA sequencing.

Several published studies have used genome-wide transcriptomics to investigate the host systemic response to TB, pathway biology and to identify diagnostic signatures to distinguish active TB from LTBI, uninfected controls, and other diseases (Berry et al., 2010; Maertzdorf et al., 2011a; Bloom et al., 2012; Kaforou et al., 2013; Anderson et al., 2014; Tientcheu et al., 2015). These studies were conducted in different geographical regions, and HIV infection status. Previous studies have reported that a comparison of independently identified differentially expressed genes and diagnostic signatures revealed that less that 80% of differentially regulated genes and only about 60% of interferon (IFN) gamma signature genes were consistently found in all studies (Maertzdorf et al., 2011a). In analyzing whole blood transcriptomics, it is critical to account for cellular heterogeneity yet an investigation of cellular proportions and pathways by applying cell-specific deconvolution algorithms remains lacking for current TB expression profiling studies. Moreover, independent TB transcriptomics studies conducted for adults and children can be further complicated by the different clinical manifestations, prior history of infection, and for these reasons maybe anticipated to have different systemic gene expression signatures. In this connection, it is worth noting that a comparison between adult and childhood TB expression profiles has not been reported. Moreover, given the wide age range, population, and cellular heterogeneity of these large data-rich studies, it is not too surprising that the clinical usefulness of expression signatures remains uncertain.

Meta-analyses can provide a statistically stringent and powerful approach to integrate and computationally deconvolute large independent datasets and thereby infer with high confidence new and consistently differentially expressed genes and pathways across multiple studies and age groups. Very recently, several studies have conducted a meta-analysis of a subset of publicly available TB transcriptomic studies; however, none of these meta-analyses studies involved a complete and comprehensive range of both adult and childhood TB (Sambarey et al., 2017; Wang et al., 2018) Most notably, the primary data in these studies have yet to be used to identify sentinel immune-metabolic pathways and to deconvolute the proportions of different immune cell types during TB and which are critical in understanding more completely the host systemic responses and for accounting the contribution of specific immune cell type pathways to disease pathogenesis.

In the present investigation, we evaluate the primary hypothesis that a common pathogenic predictive pathway biology response (inclusive of cellular, molecular, and metabolic determinants) underpins TB across different ages and populations. Accordingly, this raises a key question of delineating high confidence qualitative and quantitative pathway biology differences between childhood and adult TB. To address the central hypothesis and question, we used meta-analysis for integrating data from heterogeneous populations, to identify high confidence differentially and similarly expressed marker genes in children and adults, with active and latent TB across multiple international observational case control studies, irrespective of HIV status, geographical location, and circulating endemic pathogens. Furthermore, we performed gene expression deconvolution to determine which cell types constituted the highest proportions in TB patients and are possible contributors to disease pathologies.

## Materials and Methods

### Data Acquisition

Gene expression data repositories (GEO and ArrayExpress) were searched for datasets on human response to TB in English for meta-analysis with the following criteria: (I) Search term; {(human TB whole blood) AND “Homo sapiens”[porgn:__txid9606]} was used to retrieve datasets relating to TB gene expression. (II) Each study should be conducted using whole blood not peripheral blood mononuclear cells (PBMCs) as PBMCs when correctly purified have little granulocytes which have been shown to contribute to *M. tuberculosis* pathogenesis (Lowe et al., 2012). (III) The study should include active TB and one of either latently infected individuals (LTBI) or uninfected controls. (IV) Studies conducted using custom arrays or using qPCR were excluded because such array does not contain all genes in the genome and could be biased for specific cell types. Furthermore, following database searches publications associated with selected datasets were read to confirm clinical definitions and whether patients were treated prior to blood collection for microarray. Where datasets were not accessible or raw datasets were not available, authors were contacted for raw datasets, but further data were not made available. All searches were done on publicly available data repositories; no unpublished abstracts and studies were considered. A second assessor role (TF and PD) refereed issues arising from primary data and for workflow accuracy with any discrepancies resolved by discussion and consensus with BK and PG. A detailed workflow of data acquisition, curation, and analysis can be found in **Supplementary Figure S1**. As defined by the original studies, whole blood for transcriptome studies was collected before initiation of treatment and in one study before or within 24 h of initiation of treatment (Kaforou et al., 2013). All searches were done on or before May 2016. While reporting guidelines have yet to be standardized for meta-analysis of gene expression, we adhered as close as possible to guidelines for the meta-analysis of observational studies in epidemiology (MOOSE, **Supplementary Table S2**).

### Quality Control, Filtering, and Summarization

The arrayQualityMetrics package in bioconductor (Gentleman et al., 2004; Kauffmann et al., 2009) was used to assess the quality of each dataset in the R statistical environment. After quality control, each dataset was independently normalized before differential expression analysis. Datasets were generated using different array platforms (Illumina, Affymetrix, or Agilent), which have different probe identifiers (IDs) representing different genes on the array chip. Therefore, probes were summarized to Entrez gene levels by selecting probes with the largest median across the samples regardless of clinical phenotype to represent a specific gene. Genes present in at least two datasets were selected for inclusion in meta-analyses to account for as many genes as possible across different array platforms. Prior to extensive statistical analysis, an exploratory analysis of pairwise correlations between fold changes of the datasets was used to identity outlier datasets that could bias the meta-analysis. Only datasets with good pairwise correlations to other datasets were selected for downstream analysis.

### Differential Expression Statistical Analysis

To determine genes differentially expressed in active TB, dataset specific and meta-analysis approaches were performed using the RankProd package in the R statistical environment for estimation of percentage of false prediction (Hong et al., 2006; R Development Core Team, 2011). Meta-analyses for adult and childhood TB were conducted separately. For each individual study, three pairwise contrasts were made where possible; these were active TB versus LTBI, active TB versus uninfected controls, and LTBI versus uninfected controls. Second, the same comparisons were made in a rank product meta-analysis, resulting in an overall estimate of gene expression fold changes and statistical significance across all studies under consideration. The childhood studies lacked uninfected controls in the original studies; therefore, contrasts were performed with LTBI alone and assume that LTBI exhibits a less immune stimulatory status. Differentially expressed genes (adjusted *p*-value < 0.05, absolute fold change 1.5) obtained from the meta-analysis and from the individual study analysis were compared to identify with high confidence differentially expressed genes for downstream systemic pathway biology analysis. Given the fundamental differences between manifestations of TB in adults and children (Alcaïs et al., 2005), differentially expressed genes obtained from adult and childhood TB were also compared to identify genes commonly differentially expressed in both and those uniquely expressed in either patient population.

### Whole Blood Cellular Deconvolution

The cellular components of whole blood can vary in different physiological states and during infections and illness and could influence results obtained from whole blood differential expression analysis. Accordingly, the CellMix package (Gaujoux and Seoighe, 2013) was used to deconvolute the proportions of different immune cell types in active TB patients, latently infected individuals, and uninfected controls. The signatures used for the deconvolution are based on individual immune cell signatures identified based on cell specific gene expression analyses as previously described (Abbas et al., 2005). The normalized and log_2_ transformed dataset was used for the deconvolution using *gedBlood* function in the *CellMix* package (Gaujoux and Seoighe, 2013). Student’s *t*-test was used to perform pairwise statistical analysis of cell proportions between different clinical phenotypes in each dataset. For each cell type, the median of the controls (either LTBI or uninfected controls) was subtracted from the active TB patients for graphical representation per dataset.

### Pathway Analysis

The manually curated innate database (InnateDB; Dunkelberger and Song, 2010) was used for pathway analysis to interrogate biological pathways associated with differentially regulated genes in active TB. Up and downregulated genes were analyzed separately and for each, three separate analyses were made; genes differentially regulated (adjusted *p*-value < 0.05 and fold change > 1.5) in both adult and childhood TB, those regulated only in adults, and those regulated only childhood TB. To generate graphical representations of significant pathways, adjusted *p*-values were -log_10_ transformed and plotted on the *x*-axis for each pathway.

## Results

### Data Curation, Quality Control, and Analysis Workflow

**FIGURE 1:**
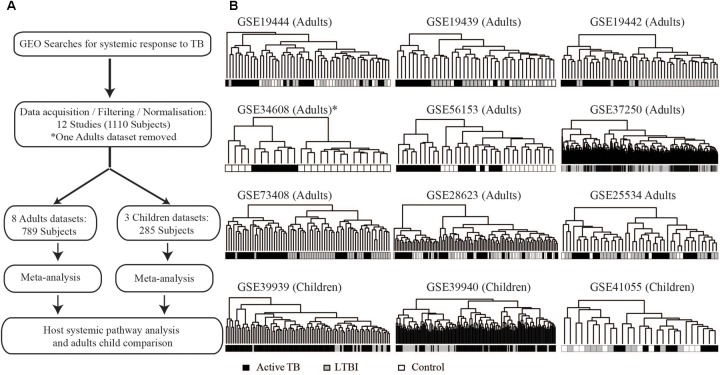
Analysis workflow and exploratory analysis. **(A)** Summary of the analysis workflow from data acquisition to pathway analysis; a detailed analysis workflow can be found in the **Supplementary Material**. **(B)** Hierarchical clustering of the top 1000 most variable gene probes largely clustering active TB from controls (uninfected controls and latently infected individuals). Pairwise comparison of FCs of different datasets can be found in **Supplementary Figure S2** indicating the outlier dataset (GSE34608).

Twelve microarray datasets with total sample size of 1110 patients (consisting of active TB patients, LTBI, and uninfected controls) passed predefined criteria. Of the 12 datasets, nine were from adults (825 patients) and three datasets were obtained from children (285 subjects). The studies analyzed here include both HIV negative and HIV positive patients as defined per the original studies. **Table 1** contains information about datasets included in this analysis and their respective GEO accession numbers. **Table 1** also shows the geographical locations of sample collections sites, year the study was conducted, array platforms used for the microarray, and the age definition of the patients. Before extensive differential expression analysis, an exploratory analysis was done on the twelve datasets, as outlined in the analysis workflow in **Figure 1A** (**Supplementary Figure S1**). In this exploratory analysis, the top 1000 most variable probes in the datasets were used to perform a hierarchical unsupervised clustering to determine if expression of these genes can cluster samples from different clinical phenotypes and identify outlier datasets. The clustering largely grouped active TB patients from LTBI and uninfected controls but there was no clear distinct clustering of latently infected individuals and uninfected controls as shown in **Figure 1B**. Pairwise correlations of the different datasets were done to identify outlier datasets. Dataset (GSE34608) was found not to be well correlated with the rest of the other datasets (**Table 1** and **Supplementary Figures S2–S4**) and was therefore removed from the differential expression analysis pipeline. Furthermore, after QC for each dataset, poor quality samples were removed. The number of samples removed for each dataset can be found in **Supplementary Figure S1** and the outlier dataset also removed resulting in 11 datasets consisting of 1073 subjects taken forward for meta-analysis.

**Table 1:** Summary of datasets included in the study.

	GEO accession number	Year	Platform	Country	Age	HC	Latent TB	Active TB	Comments and (ref)
1	GSE19439	2010	Illumina GPL6947	UK	Adults	12	17	13	Batch corrected and merge datasets from same study and site Berry et al., 2010
2	GSE19444	2010	Illumina GPL6947	UK	Adults	12	21	21	
3	GSE19442	2010	Illumina GPL6947	South Africa	Adults	NA	31	20	Berry et al., 2010
4	GSE25534	2010	Agilent GPL1708	South Africa	Adults	9	34	33	Maertzdorf et al., 2011b
5	GSE28623	2011	Agilent GPL4133	Gambia	Adults	37	25	46	
6	GSE34608	2012	Agilent GPL6480	Germany	Adults	18	NA	8	Excluded; low correlation with other datasets Maertzdorf et al., 2012
7	GSE37250	2013	Illumina GPL10558	South Africa/ Malawi	Adults	NA	167	195	Kaforou et al., 2013
8	GSE56153	2012	Illumina GPL6883	Indonesia	Adults	18	NA	18	Ottenhoff et al., 2012
9	GSE73408	2016	Affymetrix GPL11532	USA	Adults	NA	35	35	Walter et al., 2016
10	GSE39939	2014	Illumina GPL10558	Kenya	children	NA	14	79	Anderson et al., 2014
11	GSE39940	2014	Illumina GPL10558	South Africa/ Malawi	children	NA	54	111	Anderson et al., 2014
12	GSE41055	2013	Affymetrix GLP5175	Venezuela	children	9	9	9	Verhagen et al., 2013

*TB, tuberculosis; NA, not available; GEO, Gene Expression omnibus. The year column shows the year the study was done, the platform column is microarray platform used for arraying the samples, country column shows the countries from which the samples were collected, age column is the age category for the patient, and the comment and (ref) column contains the reference and comments where possible if the dataset was remove or not and what other process was done on the datasets*.

### Host Systemic Perturbation in Active Tuberculosis: Re-analysis of Individual Studies

After normalization and probe summarization across datasets, 18,945 Entrez IDs in adults and 18,629 Entrez IDs in children passed filtering. These gene probes were subjected to standardized re-analysis for differential expression analysis for individual studies and meta-analysis across all studies using the RankProd package. **Tables 2A–C** show a summary of differentially expressed genes obtained in the individual dataset analyses showing that there were more upregulated genes than downregulated genes, which could be due to increase immune modulation in response to the infection.

**Table 2:** Number of differentially expressed genes from individual dataset analysis.

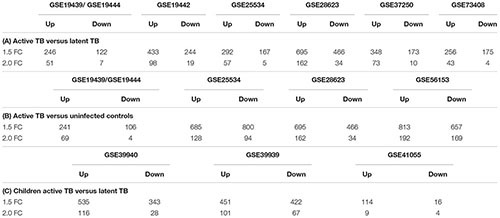

In the active TB versus LTBI comparison, GSE28623 had the largest number of differentially regulated genes while GSE19439/GSE19444 had the smallest number of differentially expressed genes as shown in **Table 2A**. Comparing active TB with uninfected controls, the same datasets had the smallest and the largest number of differentially regulated genes, respectively, as shown in **Table 2B**. In active TB versus LTBI, only 81 genes were differentially expressed across all datasets at 1.5-fold change cut-off and 66 genes were differentially regulated across all datasets in active TB versus uninfected controls (**Table 3**). This outcome underscores the magnitude of variability in transcriptional responses in these patient population studies and which could be due to several factors including sample preparation, array processing, and circulating endemic pathogens in different geographical locations. Therefore, to account for different factors affecting different datasets, a combined meta-analysis based on a rank estimate with permutation was used to identify consistently differentially expressed genes.

**Table 3:** Genes differentially regulated across all studies in the study specific analyses.

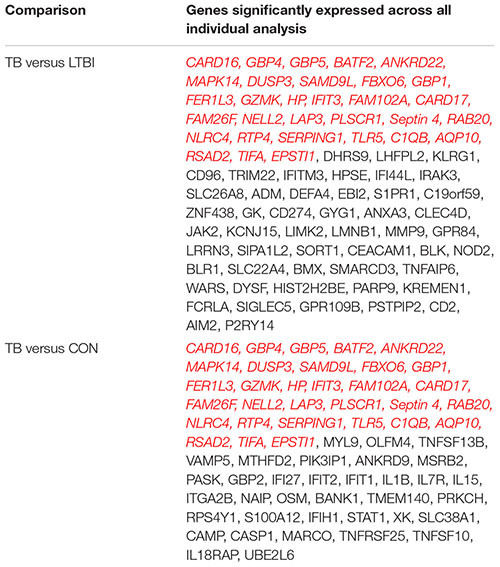

*Red and italicized are regulated in both comparisons*.

Quantitatively children developed more differentially expressed genes compared to adults as shown in **Table 2C**. However, it is noteworthy that the amplitude of the per gene fold change differences between adults and children was comparable. High variability is also notable in the childhood studies with dataset GSE41055 yielding the smallest number of differentially regulated genes with only nine upregulated and four downregulated genes at a twofold change cut-off. The two African datasets had similar numbers of differentially expressed genes. Overall, there were more upregulated genes than downregulated genes (**Table 2**) in both adults and children.

### Meta-Analysis Identified High Confidence Differentially Expressed Genes Across Datasets

**FIGURE 2:**
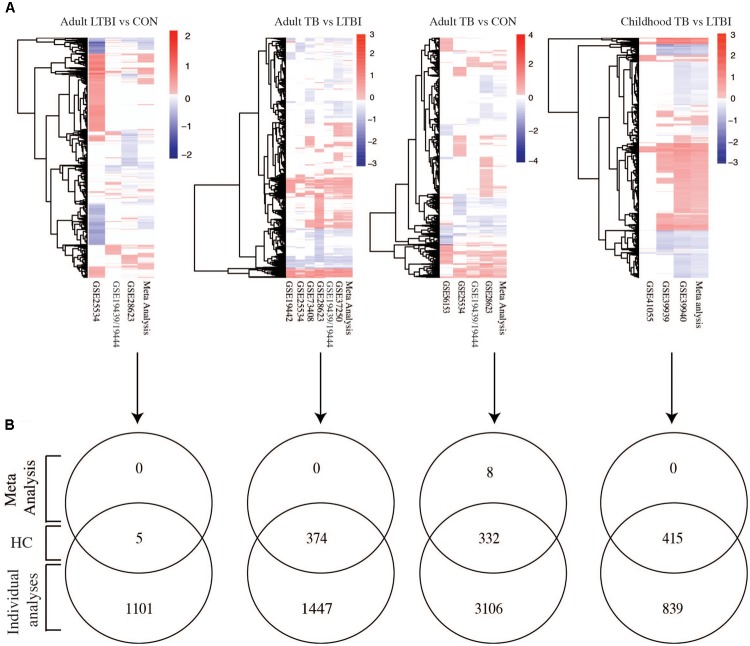
Meta-analysis identified highly robust confidence genes. **(A)** Heatmaps showing fold changes of differential expression obtained from meta-analysis using the RankProd and individual dataset specific analysis. **(B)** Venn diagrams comparing genes obtained from meta-analysis and individual dataset specific analysis to identify high confidence genes. HC, high confidence genes, which are consistently identified by both the meta-analysis and individual dataset specific approaches. TB, tuberculosis; LTBI, latent TB infection; CON, uninfected controls. Fold change cut-off 1.5, *p*-value < 0.05.

The analysis of individual studies is subject to study design and technical biases that affect reproducibility or relevance. Therefore, to identify with high confidence genes that were reproducibly regulated across different datasets, differentially expressed genes from the meta-analysis were compared with genes from the individual dataset specific analyses. **Figure 2A** shows heatmaps of the fold changes of significant genes (adjusted *p*-values < 0.05) obtained from the meta-analysis for each phenotypic comparison made and those obtained from independently analyzing each of the datasets. As expected from a meta-analysis, it can be seen that many genes with notable fold changes in individual studies lose this characteristic in meta-analysis (where the influence of individual studies is reduced by size-based weighting and in context of all studies), thereby reducing the count of false positive results and increasing the number of high confidence genes with differential expression. Only five genes were identified as high confidence in LTBI versus uninfected controls further highlighting the gene expression similarities between these two groups (**Supplementary Table S1**). Active TB versus LTBI had 374 differentially expressed genes which were identified with high confidence while active TB versus uninfected controls had 332 genes. Active TB versus LTBI in children had more high confidence genes (415 genes) compared to adult TB (374 genes) (**Figure 2B** and **Supplementary Table S1**). Eight genes were identified by meta-analysis alone in adults with active TB versus uninfected controls and not in any individual dataset specific analysis. These are CMPK2, FCGR1A, HSPA1B, LOC440607, CCL3L1, LOC649853, LOC653980, and CCL4L2. In summary, while the meta-analysis failed to identify many new differentially expressed compared to the individual studies analyses, a highly robust high confident set of expression signals was developed with small number of previously undisclosed markers. Most notably, meta-analysis significantly reduced the number of false positive results, eliminating genes with low reproducibility in differential expression across studies. For all subsequent pathway analyses, we focused on these high confidence sets of genes.

### Overlap Between Childhood and Adult Meta-Analyses Detected Genes and Previously Identified Diagnostic Signatures

**FIGURE 3:**
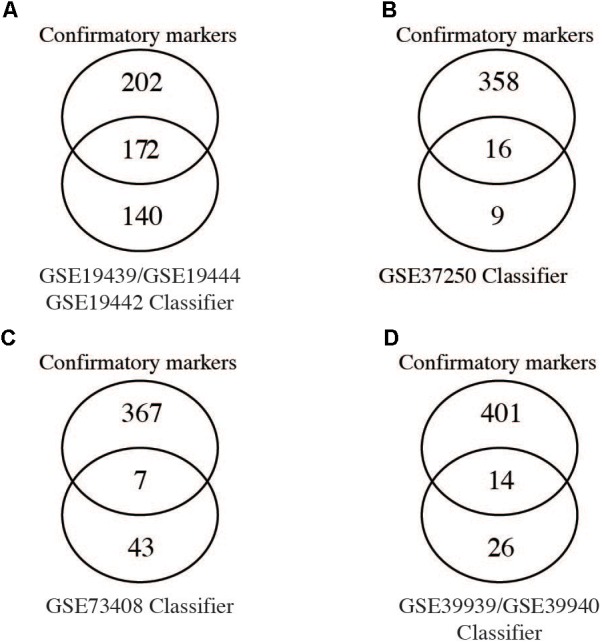
Comparison of meta-analysis to identified gene signatures. Genes significantly differentially regulated (fold change > 1.5) from the meta-analysis were compared to gene signatures identified by different studies as shown. **(A–C)** Comparison of individual diagnostics signature markers to genes from meta-analysis of adult active TB versus LTBI. **(D)** Comparison of the childhood TB signatures to childhood active TB versus LTBI.

Given that several studies have identified gene signatures potentially useable for novel TB diagnosis, we next compared differentially expressed genes (FC cut-off 1.5, adjusted *p*-values < 0.05) from the meta-analysis with gene signatures identified by previous studies. Berry et al. (2010) identified 380 transcripts (312 genes) as a TB diagnostic signature of which, only 55% (172 genes) were identified by the meta-analysis while the remaining 140 genes were not (**Figure 3A**). Kaforou et al. (2013) describe 27 transcripts (25 genes) as a classifier to diagnose adult TB, of which only 16 genes (64%) were confirmed as significant by the meta-analysis (**Figure 3B**). Another 51 transcripts (50 gene) signature was identified by Walter et al. (2016) and only 14% (Kaforou et al., 2013) of these genes were identified as confirmatory markers (**Figure 3C**). Finally, in the childhood TB diagnostic signature (42 transcript; 40 genes; Anderson et al., 2014), 33% (14 genes) were identified by the meta-analysis while the remaining 26 were not (**Figure 3D**). This reveals a disquietingly marked low confirmatory rate for the existing published expression signatures that range at best from 64% to as low as 14% identity. Our results thus identify those “unreliable” diagnostic signature markers that are likely a consequence of the heterogeneity and high variability of expression in the different populations. However, it is conceivable that the low concordance could be due to the differences in the fold change set for the meta-analysis (FC 1.5) in comparison to those set by the different studies. In Kaforou et al. (2013) and Anderson et al. (2014), the fold change was set to log_2_ 0.5 (1.4 linear fold change), and in Walter et al. (2016), 1.2 linear fold change was set during feature selection.

### Common Predictive Pathway Biology Response to Active Tuberculosis

**FIGURE 4:**
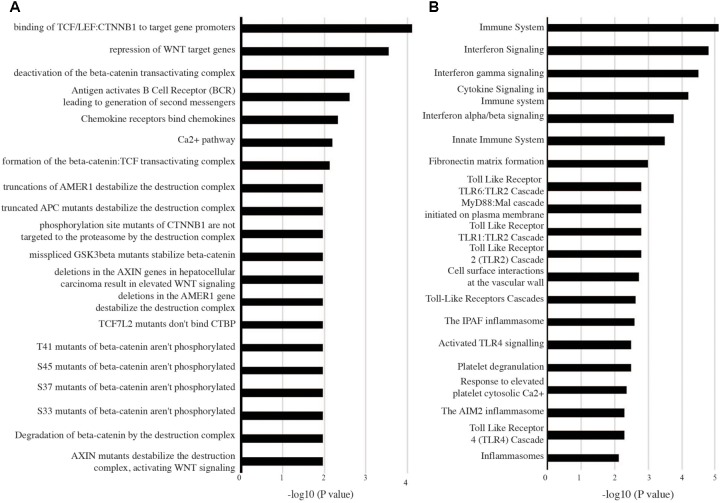
Biological pathways enriched in both adult and childhood tuberculosis (active TB versus LTBI). High confidence genes differentially expressed (1.5-fold change and adjusted *p*-value < 0.05) in both adults and children were analyzed with InnateDB to identify enriched pathways. **(A)** Pathways downregulated and **(B)** pathways upregulated in active tuberculosis.

Outputs of the meta-analyses can be further used to inform high confidence pathway analysis of active TB versus LTBI. Here, genes upregulated in both adults and children were enriched with pathways associated with innate immune responses including type I IFN alpha/beta signaling, TLR signaling, cytokine signaling, and numerous metabolic transporters and enzymes. Notably complement activation (*C1QB*, *C1QC*, *CR1*, *PROS1*), defensin (*SLPI*, *DEFA1*, *A3*, and *A4*) and antimicrobial factors (*S100A12*, *LCN2*, *DDX60L*, *LTF*, *TRRD9*, *ADM*, *APOBEC3A*), and inflammasome activation (*IL18R1*, *IL18RAP*, *CARD16* and *17*, *NLRC4*, *AIM2*, *NAIP*, *CASP1*) were all significantly upregulated (**Figure 4B**). *TLR2* signaling was the most prominent TLR pathway and included its interaction with *TLR1* and *TLR6* cascade signaling (**Figure 4B**). Other notable pattern recognition receptors equally regulated in adults and children included *TLR5*, *Ly96*, *PGLYRP-1*, and importantly *CLEC4D* and *E* that recognize the mycobacterial cell wall glycolipid trehalose 6,6′-dimycolate. Previously unrecognized innate immune pathways were also upregulated, in particular *OLM4*, WNT signaling involving *KREMEN1*, *LRRK2*, and *TSH23* and apparent alternative immunosuppressive macrophage markers including *ARG1*, *MS4ALA* and related atypical chemokine receptors *CCRL2*. Further upregulated immune suppressive signaling includes *IRAK3*, *SIGLEC5*, *SLPI*, *PSTPIP2*, *PIK3AP1*, and the lymphoid co-inhibitory molecule *CD274* (*PD-L1*).

In connection with the upregulation of multiple inhibitory pathways, genes significantly downregulated in both adult and childhood TB were predominantly enriched with pathways including chemokine receptor binding, Ca^2+^ signaling, B-cell (*CD79A* and *B*, *BLR1*, *EBI2*, *VPREB3*, *ID3*, *FCRLA*, *FAIM3*, *FCGBP*, *CCR7*) and T-cell (*BACH2*, *CD6*, *CD5*, *BCL11B*, *TCL1A*, *TCF7*, *CCR7*, *LEF1*), and T/NK cell functions that showed significant suppression of the CD3 zeta (*CD247*)-EPHA4/NCR3 axis, *CD96*, *UBASH3A*, *GZMK*, and critically *IL7R* that when suppressed leads to severe immunodeficiency (**Figure 4A**).

Thus, a common pathogenic predictive pathway biology response underpins TB across different ages and populations. In active TB, this comprises an upregulation of the inflammatory innate arm of the immune system (encompassing a marked inflammasome type I IFN-TLR2 axis, complement activation, and antimicrobial factors) and which is concomitant with immunosuppressive downregulation of the adaptive immune arm (encompassing predominantly B and T cell signaling and effector functions).

### Correlation Between Active TB Versus LTBI to Active TB Versus Uninfected Controls

**FIGURE 5:**
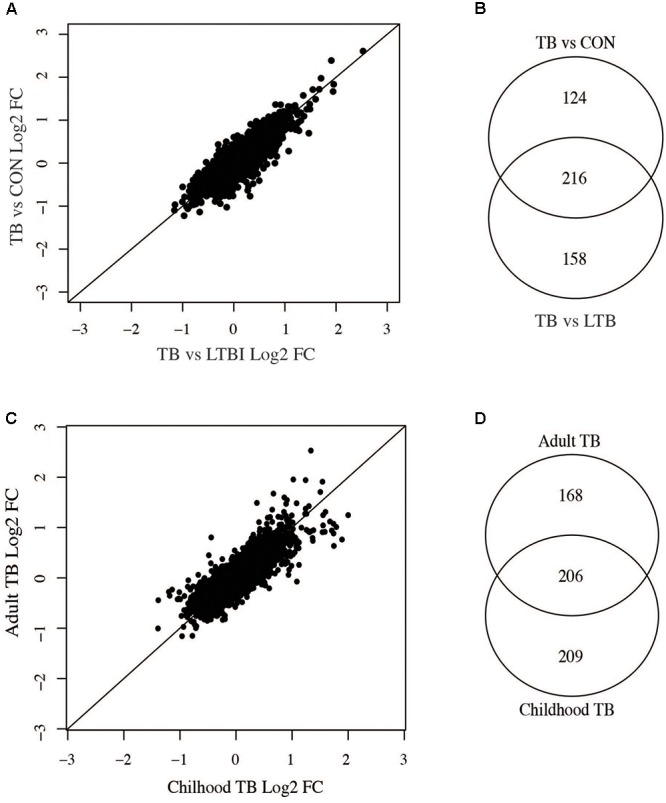
Comparisons of adult active TB versus LTBI and controls and childhood versus adult TB. **(A)** Correlation between adult active TB versus latent TB and active TB versus uninfected controls. **(B)** Number of genes common between active TB versus latent and active TB versus uninfected controls. **(C)** Correlation of fold changes obtained in active versus latent TB in children and adults. **(D)** Overlap of differentially expressed genes in adult and childhood TB.

Individuals with LTBI and uninfected controls showed similar expression profiles and could not be distinctly clustered based on the 1000 most variable genes (**Figure 1B**). We therefore compared differentially expressed genes between active TB versus LTBI and those from active TB versus uninfected individuals to identify phenotype specific gene sets. There was a good correlation of fold changes obtained from the two comparisons (**Figure 5A**) and a good overlap between differentially expressed genes at 1.5-fold change cut-off (adjusted *p*-value < 0.05) while some genes were only expressed on either of the comparisons. Two hundred and sixteen genes were common to both comparisons, 124 genes differentially expressed only in active TB versus healthy controls, and 158 only expressed in active TB versus LTBI as shown in **Figure 5B**. In summary, this suggests as expected that the lack of specific clustering of differentially expressed genes in either comparison is indicative of a less immune activated status in latent TB in comparison with active TB. However, it is also notable there are limited set of genes specific to latent TB phenotype and point to a subtle yet detectable difference.

### Comparison Between Childhood and Adult TB Host Responses

**FIGURE 6:**
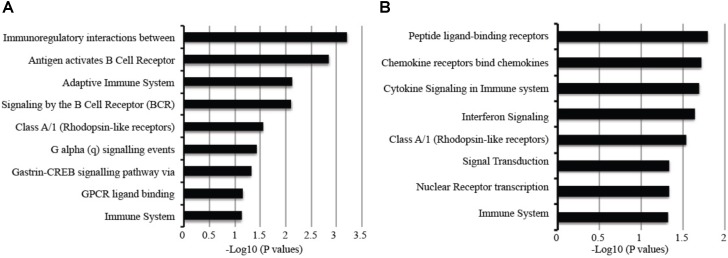
Pathways associated with gene specific to **(A)** active TB versus latent TB and **(B)** active TB versus control. Long fold change cut-off at 1.5 and *p*-value of <0.05.

Childhood and adult TB clinical features and manifestations differ; in adults, the infection is largely restricted to the lungs while in children the infection can often disseminate to other parts of the body (Alcaïs et al., 2005) and hence could result in vastly different host systemic transcriptional responses. Therefore, we compared adult and childhood TB host systemic transcriptional responses and unexpectedly found good overlap of differentially expressed genes between them with a smaller subset of genes specific to either adults or children. **Figure 5C** shows there was a good correlation of fold changes obtained from adult and childhood active TB versus LTBI. However, some genes exhibit higher fold changes in adults and vice versa as shown in **Figure 5C**. At a 1.5 FC cut-off, 206 genes were differentially expressed in both adult and childhood TB, 168 differentially expressed only in adults, and more genes (209 genes) were differentially expressed only in childhood TB (**Figures 5C,D**) and their associated pathways are shown in **Figure 6**. In summary, we observed that childhood TB has about 20% more systemic differentially expressed genes than adults but shared the same differential pattern (representing over 55%) of the differential expressed genes in adult TB.

Nevertheless, genes could be identified that were significantly upregulated only in adult TB represented an extension of the common pathways noted above associated with innate immune inflammatory response including an increased number of IFN regulated genes (*PML*, *CXCL10*, *IFITM1*, *IRF7*, *OAS1*, *IFITM4P*, *ISG15*, *IFI35*, *STAT1*, *SOCS1*, *DUSP3*), *TLR4*, *TLR7/8*, and *TLR9* signaling cascades, and a further augmented significance for the first regulated step of the glycolytic pathway (HK3).

The adult selective downregulated gene signatures accentuate the suppressed adaptive immune pathways including T-cell cytotoxic granulysin molecule (GNLY) against TB, TCR alpha/beta or gamma delta signaling (in particular *CD2*, *EOMES*, *TRA*, *CD8A*, *TRBV5-4*, *ZAP70*, *SKAP1*, *CD3D*, *TARP*, *FGFB2*, *RASGRP1*, *DYRK2*, *EVL*, *SH2D1A*, *ITK*, *MATK*), B-cell functions (*BANK1*, *CD19*, *BLK*, *FAM129C*, *MS4A1*, *FCRL3*, *EBF1*, *SPIB*, *RASGRP1*, *SH2D1A*, *FCRL2*). NK/T-cell co-inhibitory molecules *KLRG*, *KLRB1*, and cytokine *IL32*; suppression of TGFbeta inhibitory-molecule *TGFBR3* (**Supplementary Figure S7**).

Signature sets differentially upregulated only in childhood TB were notably replete of IFN regulated genes but enriched with other immune pathways including elevation of alternative antimicrobial defense mechanisms (*H2AFJ*, *HISTIH2BG*, and *CTSG*) and multi-functional *IL27* that blocks *IL-17* and *IRF1* signaling as well as counter T-reg functions, platelet adhesion, and metabolic pathways involved in transport of glucose and other sugars, erythrocytes uptake of carbon dioxide and release of oxygen and cell surface interactions at the vascular wall. There is a significant increase in pathways associated with metal ion homeostasis including *SELENBP1*, *MT2A*, *STEAP4*, *S100P*, and cell adhesion and interactions (notable markers are *GCA*, *ESAM*, *GPR97*, *COL17A1*, and *PSG3*). There are concurrent blockade and stimulatory pathways for IFN/MDA5 activity involving the RIOK3 kinase and DDX60 pathways, respectively. While inhibitory pathways for B-cell activation (via *SAMSN1*) and leukotriene-B (Bruchfeld et al., 2015; degraded by *CYP4F3*) for polymorphonuclear leucocyte chemoattractant activity are unchecked, elevation of *PSG9* that promotes T-reg functions is countered via increased *IL27*.

Notable downregulated genes specific for childhood TB are enriched with pathways mainly involved in stimulation of T/B cells (*CD40L*, *CD7*, *ICOS*, *FCER2*, *PTPRCAP*, *ADAM23*), dendritic cell development (*FLT3LG*), alternative promoters of inflammation (*CD248* and *EDAR*), and inhibition of neutrophil degranulation by *ADORA3*. Moreover, there is a significant suppression of *IL23a* that heterodimerizes with *IL12B* to activate the JAK-STAT-IFNG axis, and the OLIG2-SIGLEC8 eosinophil-axis is low in active but high in latent TB. Further eukaryotic translation such as translation initiation, elongation and termination, metabolism of proteins, amino acid transport, and gene expression as further shown in **Supplementary Figure S7**.

In summary, a comparison between adult and childhood TB shows that over a third of the confirmatory response is shared across the different populations while the other third, significant genes are exclusively significant for either children or adult responses. Unexpectedly, our findings reveal a differential yet plastic response, showing a more significant qualitative and quantitative contribution of both type I and II IFN responses in adults over children and the use of different but functionally similar co-inhibitory molecules underscored by the upregulation of *HLA-G* and *PDCDILG2* (*PD-L2*) in children and adults, respectively.

### Active Tuberculosis Patients Present With Higher Proportions of Innate Immune Cells and Lower Proportions of Adaptive Immune Cells Compared to Controls

**FIGURE 7:**
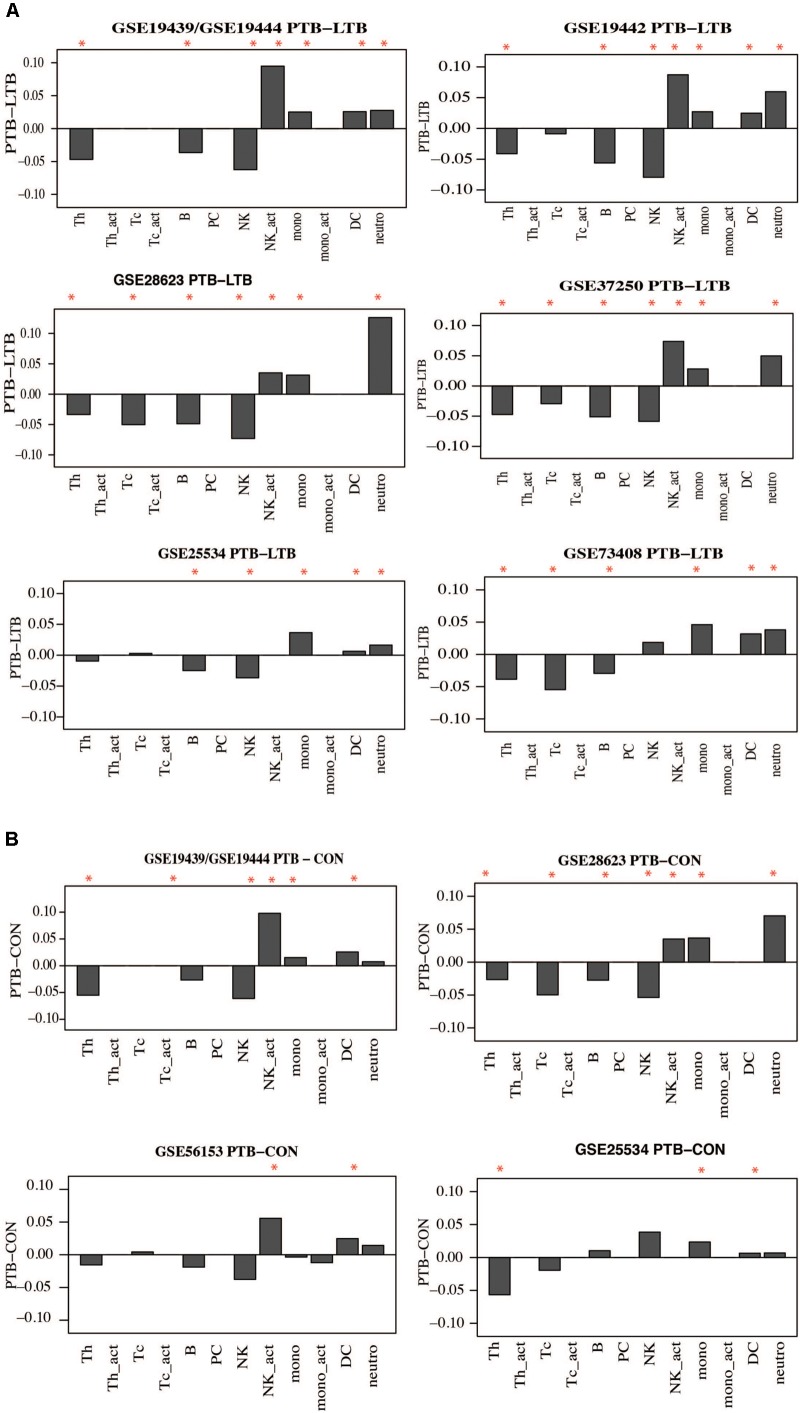
Difference in Immune cell proportions in adult tuberculosis. Gene expression signals were used to deconvolute cell proprtions in whole blood collected from TB and controls using the CellMix package. The median for each cell component per clinical phenotype was determined. Median cell proportions from controls were substracted from medians from active TB. **(A)** Immune cell proportion difference between active TB and latent TB. **(B)** Difference in active TB and uninfected controls. Red asterisks indicate those that are siginificant (*p* < 0.05) based on a Student’s *t*-test.

Whole blood is made up of different innate and adaptive immune cellular components which can change in response to internal and external challenges, such as inflammatory and infectious diseases including TB. As such, it could be expected that some immune cellular components will be different between people with active TB and LTBI and uninfected controls and could contribute to observed gene signatures. Therefore, we performed whole blood cell deconvolution to determine differences in cell proportions in active TB, LTBI, and uninfected controls using the CellMix package (Gaujoux and Seoighe, 2013). There were higher proportions of innate immune cells in active TB compared to both latently infected and uninfected individuals. Specifically, proportions of neutrophils and monocytes were significantly higher in active TB compared to controls whereas T-helper (CD4) cells and B cells were higher in controls compared to active TB patients. **Figure 7A** and **Supplementary Figure S5** show differences in cell proportions between active TB and LTBI in adults and **Figure 7B** shows cell proportion differences between active TB and uninfected controls. Resting natural killer (NK) cells were significantly lower in active TB patients while the proportions of activate NK cells were significantly higher in active TB patients. No significant differences in immune cell proportions were observed between LTBI and uninfected controls.

**FIGURE 8:**
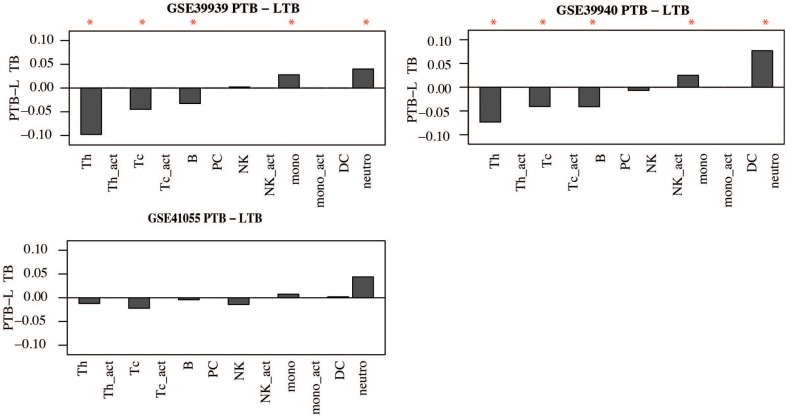
Immune cell proportions in childhood TB. Immune cell proportions were deconvoluted from whole blood based on gene expression using CellMix. Median cell proportions of latent TB patients were subtracted from the median proportions from active TB for each cell in each study. Red asterisks indicate significant differences based on Student’s *t*-test.

Similar observations were made in childhood TB; children with active TB had higher proportions of neutrophils and other innate immune cells while the proportions of B and T-cells were lower compared to latently infected children. However, significant differences in cell proportions were not observed in the childhood TB dataset collected from the Warao Amerindian children in Venezuela as shown in **Figure 8** and **Supplementary Figure S6**.

In summary, this analysis suggests that active TB patients have significantly higher proportions of innate immune cells and lower proportions of adaptive immune cells compared to controls. These alterations in cell proportions are in good agreement with alterations observed at the molecular functional pathway level involved in the pathogenesis of TB.

### Modulation of Co-inhibitory and Co-stimulatory Signaling Molecules in Tuberculosis

**FIGURE 9:**
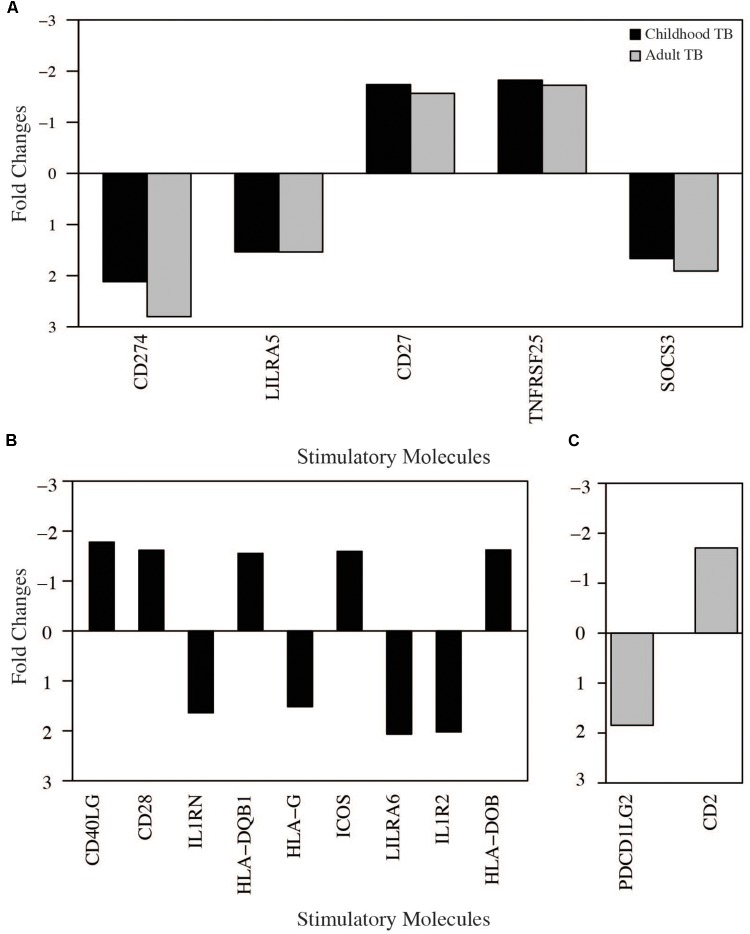
Immune cell signaling molecules modulated in tuberculosis. Significantly regulated immune cells stimulatory and inhibitory molecules obtained from tuberculosis in the meta-analysis (fold change > 1.5 adjusted *p*-value < 0.05). **(A)** Molecules modulated in both adults and childhood tuberculosis. **(B)** Molecules modulated only in childhood tuberculosis. **(C)** Molecules modulated only in adult tuberculosis.

To understand further the relationship between cellular and molecular pathway biology responses, we next investigated the modulation of host co-inhibitory and stimulatory immune signaling molecules in TB. CD 27 (*CD27*), *CD274*, and suppressor of cytokine signaling 3 (SOCS3) were upregulated while *LILRA5* and *TNFRSF25* were downregulated, respectively, in both adult and childhood TB (**Figure 9**). *CD27* is required for generation and maintenance of long-term memory by transducing the signal to activate the *NF-kB* and *MAPK8/JNK*, *CD274* encodes an immune inhibitory signal expressed in immune cells including T and B-cells, which inhibit T cell activation and cytokine production and *SOSC3* also negatively regulates immune signaling through inhibition of *JAK2* kinase. *LILRA5* and *TNFRSF25*, which were downregulated, are also important in signal transduction and generation of a strong immune response.

Only two co-stimulatory genes; *PDCD1LG2* and *CD2* were up and downregulated, respectively, exclusively in adults (**Figure 7**). *CD2* is a cell surface receptor important for antigen presentation and *PDCD1LG2* encodes a programmed cell death 1 ligand 2.

More immune stimulatory molecules were modulated in childhood TB; four upregulated (*HLA-G*, *LILRA6*, *IL1RN*, and *IL1R2*) and five downregulated (*CD40LG*, *HLA-DOB*, *CD28*, and *HLA-DQB1*). *HLA-G* encodes histocompatibility antigen, class 1G and *LILRA6* encodes for leukocyte immunoglobulin like receptor 6, both of which are important immune regulators of interaction between lymphoid and non-lymphoid cells. Importantly, *HLA-G* is known to play a critical role for suppressing maternal immunity in fetal development. *IL1RN* encodes an *IL1* receptor antagonist that inhibits *IL1* mediated signaling during the immune response and *IL1R2* is a decoy receptor which binds to IL1 alpha/beta inhibiting its activity with its ligand. *CD40LG* is a *CD40* ligand expressed on the surface of T cells and involved in the regulation of B cell function. *HLA-DOB* and *HLA-DQB1* are members of the major histocompatibility complex important for antigen processing and presentation. *CD28* is also expressed on the surface of T cells and important for T-cell proliferation, cytokine production, and Th2 cell development. Overall this data indicate upregulation of inhibitory immune signaling molecules and downregulation of co-stimulatory signaling molecules important for immune signaling during active TB.

## Discussion

In the present study, we used a systematic meta-analysis approach to comprehensively reanalyze publicly available microarray datasets to identify high confidence differentially regulated signatures in adult and childhood TB across multiple continents and a spectrum of circulating endemic pathogens and HIV infection status. Compared to the individual analyses of each dataset, the meta-analysis provided increased power to detect only a limited number of differentially regulated genes but most critically identified high confidence differentially regulated gene sets, reducing a large number of false positive markers detected in the individual study analyses. This demonstrated a small overlap of potential biomarkers across multiple studies conducted in different geographical locations, and which agrees with another very recently published meta-analysis of TB (Sambarey et al., 2017). In adults with active TB versus LTBI, 374 genes were differentially regulated and in active TB versus uninfected controls, 332 genes were differentially regulated. As observed in previous studies, the expression profiles of latently infected individuals and non-infected controls were very similar (Maertzdorf et al., 2011a) but critically identifying for the first time five significantly latent specific biomakers. When we compared genes regulated in active TB versus LTBI or active TB versus uninfected controls, there was a good overlap (216 genes). By contrast, 124 genes and 158 genes were only regulated in active TB versus uninfected controls and active TB versus LTBI, respectively. This therefore clearly indicates a much-reduced immune altered state in latent TB but which retains a minimal detectable systemic response. In relation to age, childhood TB developed a more differentially regulated genes (415) than to adult active TB versus LTBI of which approximately half (206) were also significant in adults, revealing a common core pathogenic network response as well as the anticipated differences in response to TB in adults and children that likely relate to the different clinical manifestations (Alcaïs et al., 2005).

A comparison of confirmatory differentially regulated genes with previously identified TB diagnostic signatures (Berry et al., 2010; Kaforou et al., 2013; Anderson et al., 2014) showed a low confirmatory rate (ranging from 14 to 64%) as shown in **Figure 3**. While this could be possibly explained by the different fold change cut-off set used in the independent studies, the meta-analysis approach reduces false positives and therefore the low correspondence most probably reflects the underlying heterogeneity and overall poor reproducibility for those markers. This highlights the urgent need for potential diagnostic markers to be validated across different and larger patient sample size populations, and critically the standardization of statistical methods and significant cut-offs across studies.

Pathway analyses indicated that genes up and downregulated in both adult and childhood TB were enriched with innate and adaptive immune response pathways, respectively, suggesting strong upregulation of innate inflammatory immune responses and downregulation of adaptive responses, which corroborates previous findings (Berry et al., 2010; Maertzdorf et al., 2011a). Adults show more IFN driven innate immune pathways and downregulated adaptive pathways. On the other hand, childhood specific upregulated genes were associated with inflammasome IL1RN–IL1R2 suppression axis, glucose transport, CO_2_ and O_2_ release, and cell surface interaction pathways while downregulated genes were associated with mRNA translation, protein metabolism, and amino acid transport. Indeed, recent studies have indicated that *M. tuberculosis* imports host amino acids and relies on them to thrive in the host (Gouzy et al., 2014). Most importantly, in childhood TB, the immune inhibitory molecules, *IL1RN* and *IL1R2* which inhibit functional *IL1* signaling and molecules involved in generation of an adaptive immune response (*CD40LG*, *HAL-DOB*, *CD28*) that were downregulated is consistent with emerging evidence linking the cross talk between *IL1* and type 1 IFN to TB and which provide potential targets for host directed therapy (Mayer-Barber et al., 2014). This could explain the observation that children have less pronounced adaptive immune responses to TB compared to adults, potentially resulting in disseminated forms of the diseases seen more frequently in childhood TB. Our findings further highlight the differences between adult and childhood TB and most critically a previously unrecognized potential of using the same common host signature for novel diagnostics in both adult and childhood TB.

Furthermore, whole blood deconvolution indicated high proportions of neutrophils in active TB compare to latently infected individuals and uninfected individuals which is reflective of observed pathway biology. This corroborates previous studies indicating the important role of neutrophils in TB, previously identified as major contributors to observed gene expression signatures (Martineau et al., 2007; Berry et al., 2010; Eum et al., 2010; Mcnab et al., 2011). This meta-analysis which combined studies from different locations further strengthens evidence for the important contribution of neutrophils in TB. Proportions of monocytes and activated NK cells were also higher in active TB compared to controls, and NK cells have been shown to play an important role in combating mycobacterial infections (Allen et al., 2015; Choreño Parra et al., 2017). However, proportions of resting NK cells were lower in active TB compared to LTBI individuals and uninfected controls which agrees with previous studies (Kee et al., 2012). On the other hand, proportions of T and B cells which make up the adaptive arm of the immune system were downregulated, which corroborated results observed in the pathway analysis. This is in line with recent studies showing that patients with active TB have reduced frequency of circulating B cell and T cell responses and higher in successfully TB treated individuals compared to patients recently diagnosed with TB (Joosten et al., 2016).

## Conclusion

By using meta-analysis to re-analyze publicly available microarray datasets, we identified with high confidence signature genes differentially expressed across studies removing a large number of false positive markers. Most importantly, from translational perspective, we reveal a previously unrecognized potential of using a common host immune-metabolic signature network as a novel diagnostical tool for use in both adult and childhood TB. We believe that the use of the confirmatory expression signatures identified here will help in guiding optimized diagnostic, prognostic, and therapeutic monitoring modalities for TB patients, but which will still necessitate high quality prospective randomized trials to assess clinical efficacy.

## Ethics Statement

This was meta-analysis of already existing publicly available online dataset from gene expression data repositories.

## Author Contributions

SB and PG conceived and designed the study. SB conducted the analyses. TF and PD helped with analysis. SB, BK, and PG wrote the manuscript.

## Conflict of Interest Statement

The authors declare that the research was conducted in the absence of any commercial or financial relationships that could be construed as a potential conflict of interest.

## Abbreviations

CD: cluster of differentiation
GEO: gene expression omnibus
HIV: human immunodeficiency virus
IL1: interleukin 1
TB: tuberculosis
TLR: Toll-like receptor.
