# Supplementary material for: Meta-Analysis Identification of Highly Robust and Differential Immune-Metabolic Signatures of Systemic Host Response to Acute and Latent Tuberculosis in Children and Adults
Source: Front Genet. 2018 Oct 4;9:457. doi: 10.3389/fgene.2018.00457 (PMC6180280; doi:10.3389/fgene.2018.00457)
Supplement: Supplementary file 3 [file Data_Sheet_1.pdf]

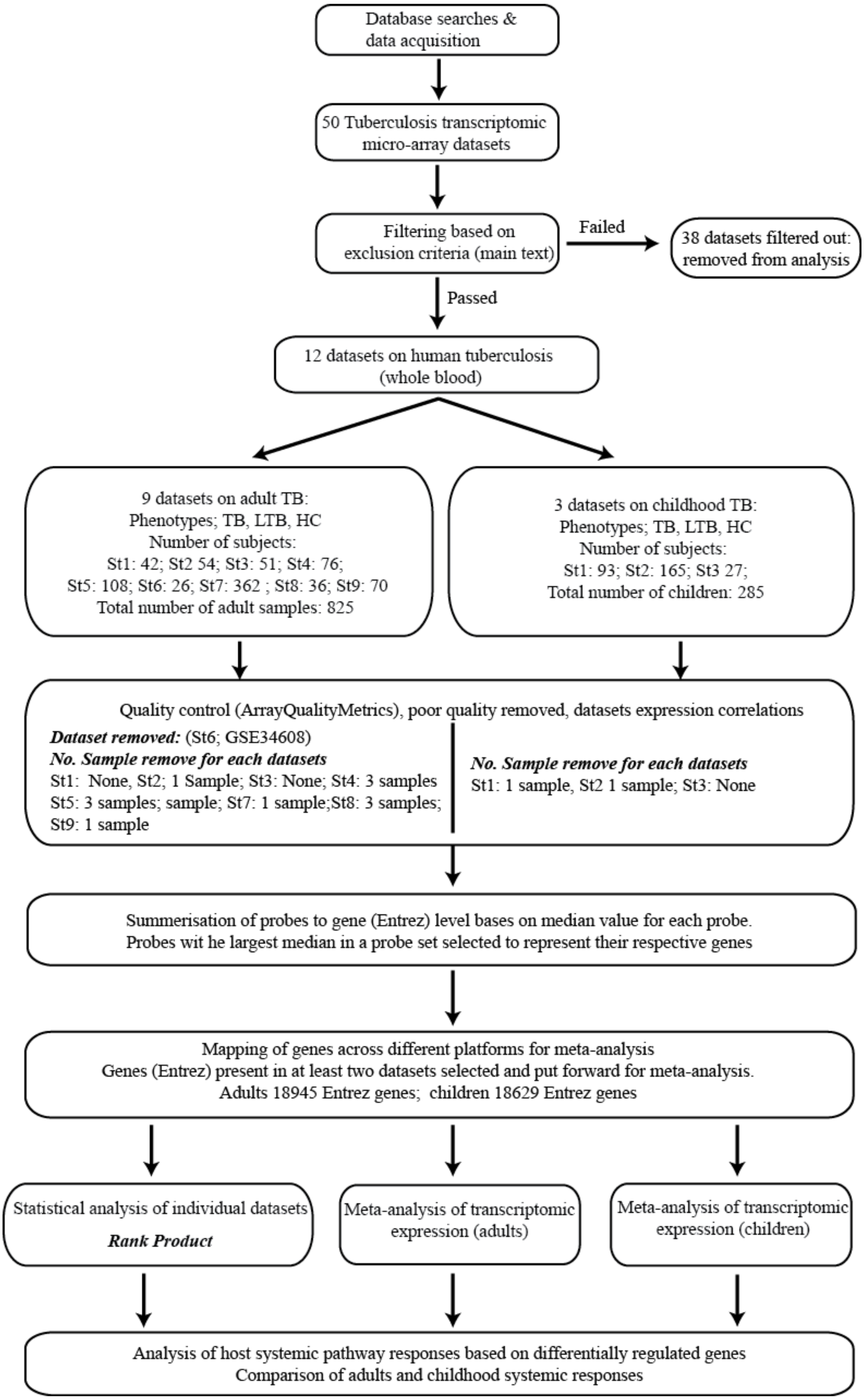

Fig S1: Data acquisition and analysis pipeline. This shows a schematic representation of the data analysis workflow, from database searches, data curation and quality control to differential expression and pathway analysis. Number of patients in each patient group is also indicated.

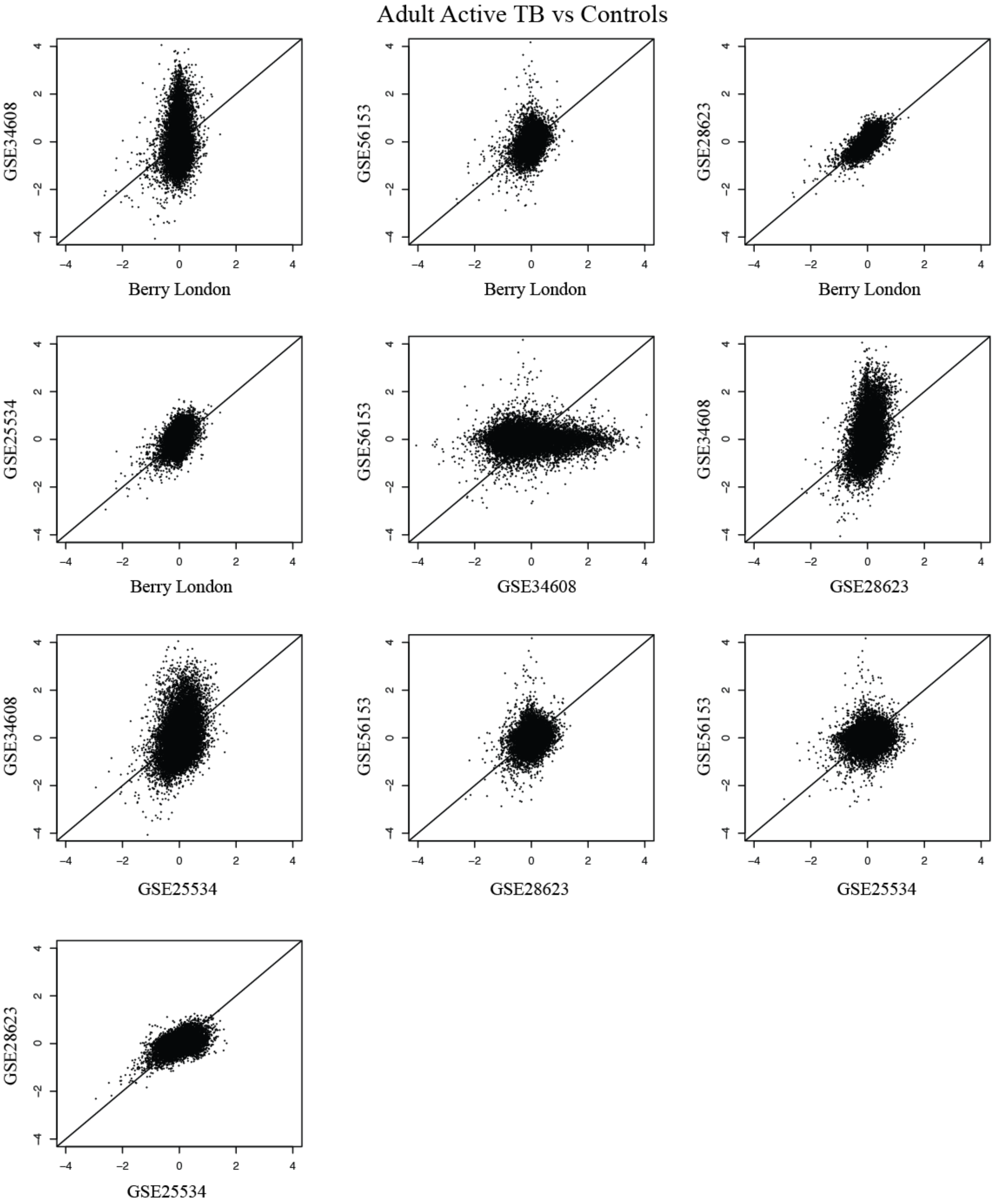

Fig S2: Pairwise correlations of the different tuberculosis datasets for adults active TB versus controls. The fold changes obtained from datasets were plotted against one another and diagonal drawn to see how well each two datasets fitted around the diagonal. GSE34608 did not correlate with most of the datasets and was therefore remove from the analysis.

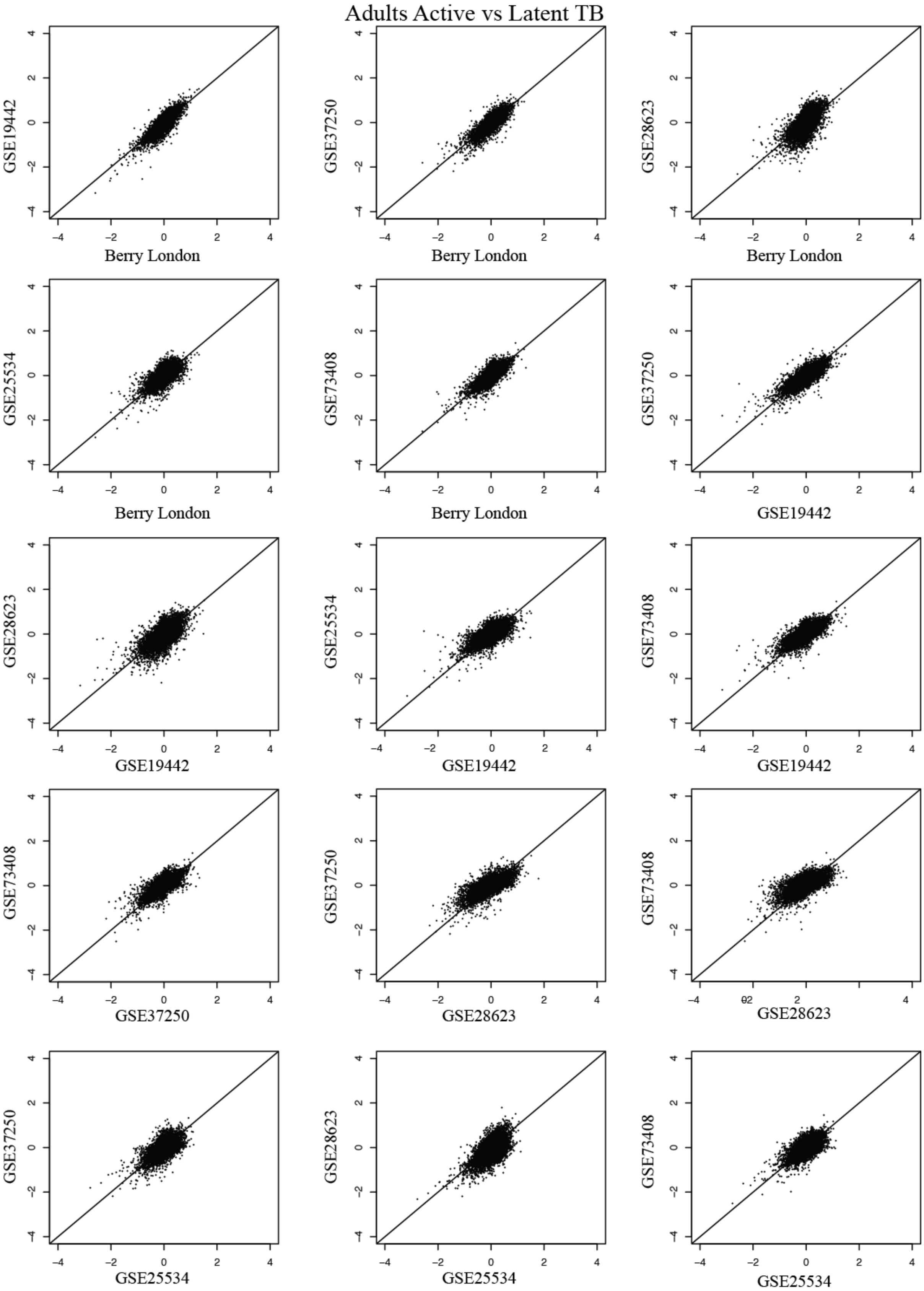

Fig S3: Pairwise correlations of the different tuberculosis datasets for adult active TB versus latent TB. Fold changes of active TB versus latent TB of each datasets plotted against other datasets.

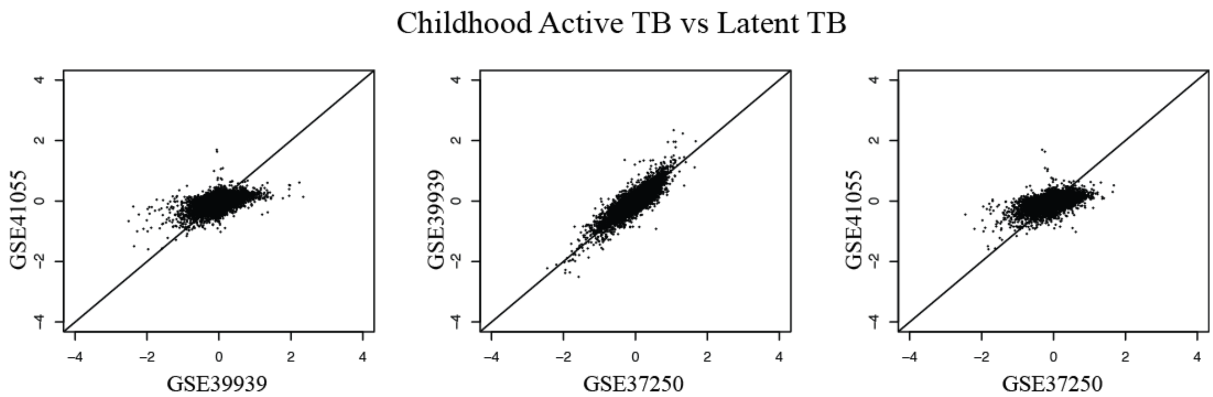

Fig S4: Pairwise correlations of the different tuberculosis datasets for childhood active TB versus latent TB. Fold changes for active TB versus latent infection for each dataset was calculated and plotted against other datasets.

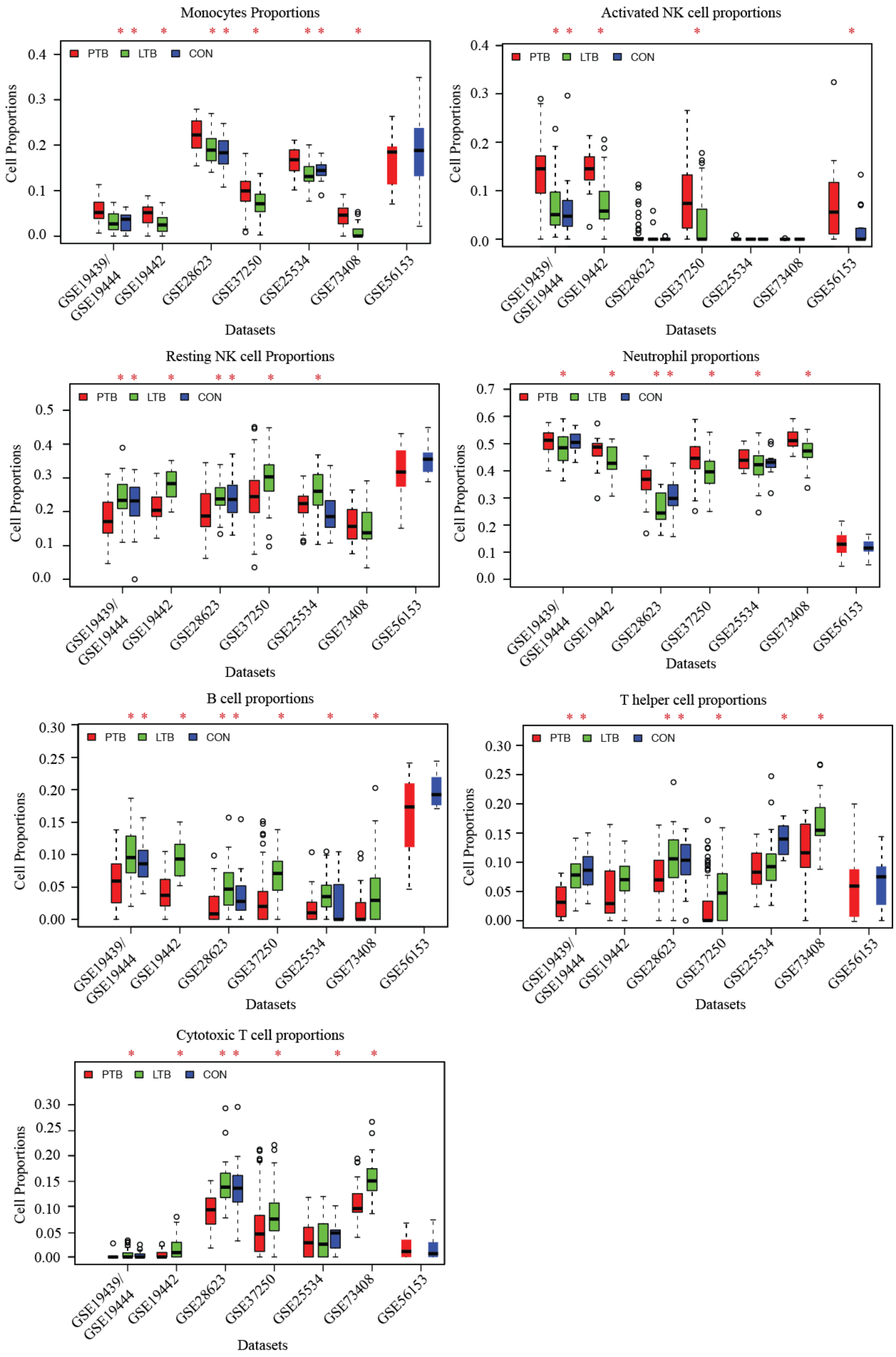

Fig S5: Box plots of different cell proportions from adult whole blood deconvolutions. Normalised and log2 transformed data for each dataset was used to find cell proportions using the CellMix package and proportions used to generate the boxplots. T-test was used to find significant difference between clinical phenotypes. The red asterisks represent significant difference (p value < 0.05).

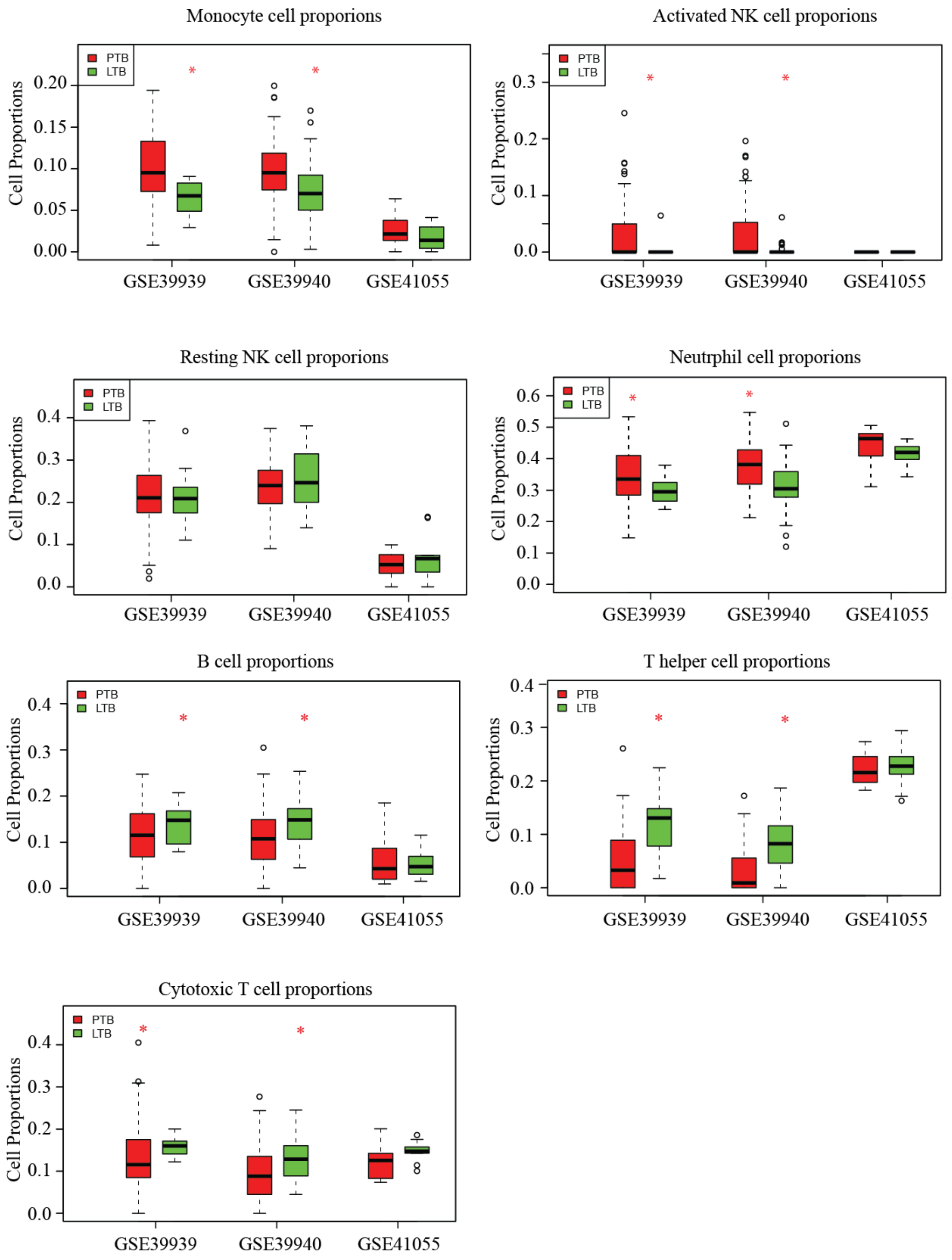

Fig S6: Box plots of different cell proportions from childhood TB whole blood deconvolution. Childhood TB datasets were normalised and log_2 transformed and the CellMix package used to calculate cell proportions and boxplot generated. T-test was used to find significant difference between different clinical phenotypes. Red asterisks indicate significant difference (p-value < 0.05)

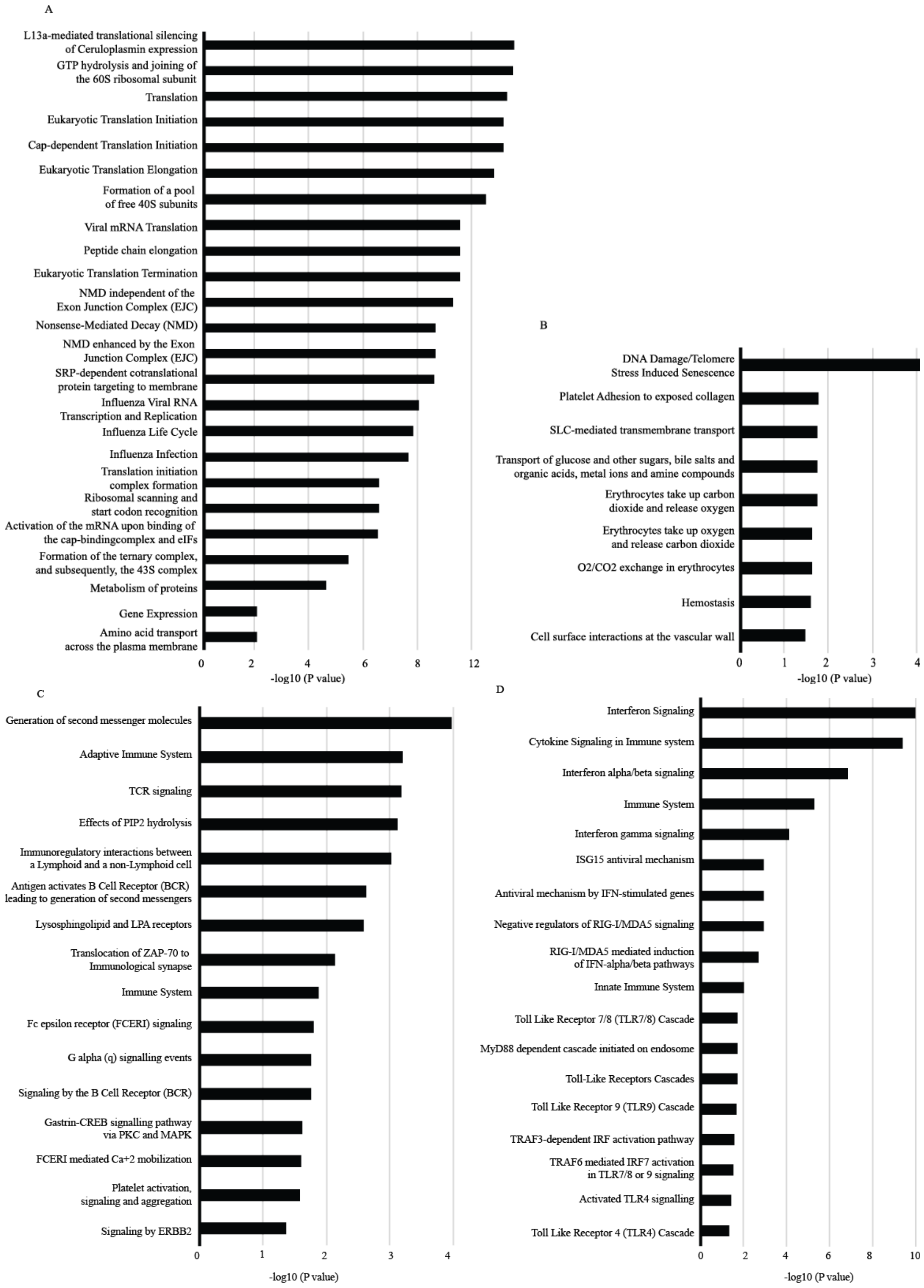

Fig S7: Pathway enriched in genes specific in adult and childhood tuberculosis. Gene specifically differentially expressed (adjusted p value < 0.05, fold change 1.5) in only in adult and childhood TB were analysed for enriched pathway using InnateDB. A) Pathways enriched in genes down regulated only in childhood TB; B)

Pathways enriched in genes up regulated only in childhood TB; C) Pathways enriched in genes down regulated only in adult TB and D) Pathways enriched in genes up regulated only in adult TB.

Abbreviation of cell types in the deconvolution

Cell specific signatures as defined by Abbas et al (1,2) were used for gene expression deconvolutions in the *CellMix* package. Below are the abbreviations of the for the cells types and stimulation condition which can be found in (1).

Th;	CD4 T-helper cell
Th_act;	activated CD4 T-helper cell
Tc;	CD8 cytotoxic T cell
B;	B cell
B_act;	activated B cell
B_aIgM;	IgM producing B cell
Mem_IgG	IgG memory cell
Mem_IgM	IgM memory cell
PC;	Plasmacytoid cell
NK;	Natural Killer cell
Mono;	Monocytes
Mono_act;	Activate monocyte
DC;	Dendritic cell
DC_act;	activated dendritic cell
Neutron;	Neutrophil

Reference:

1. Abbas AR, Baldwin D, Ma Y, Ouyang W, Gurney A, Martin F, et al. Immune response in silico (IRIS): immune-specific genes identified from a compendium of microarray expression data. Genes Immun [Internet]. 2005;6(4):319–31. Available from: http://www.ncbi.nlm.nih.gov/pubmed/15789058
2. Abbas AR, Wolslegel K, Seshasayee D, Modrusan Z, Clark HF. Deconvolution of blood microarray data identifies cellular activation patterns in systemic lupus erythematosus. PLoS One [Internet]. 2009;4(7):e6098. Available from: http://www.pubmedcentral.nih.gov/articlerender.fcgi?artid=2699551&tool=pmcentrez&rendertype=abstract
